# URBAN AGING RESIDENTS COALITION (UARC): A HYBRID MODEL TO BRIDGE THE TECHNOLOGY GAP

**DOI:** 10.1093/geroni/igac059.1660

**Published:** 2022-12-20

**Authors:** Kathy Wright, Karen Moss, Judith Tate, Karen Rose, Pamela Shields

**Affiliations:** The Ohio State University College of Nursing, Columbus, Ohio, United States; The Ohio State University, Columbus, Ohio, United States; Ohio State University, College of Nursing, Columbus, Ohio, United States; The Ohio State University, Columbus, Ohio, United States; Urban Aging Residents Coalition, Columbus, Ohio, United States

## Abstract

The COVID-19 pandemic spotlighted technology barriers for urban Black/African American older adults that lead to chronic stress, social isolation, and inability to fully engage with the world. In partnership with an African American older adult community leader, the Urban Aging Residents Coalition was founded in May 2020 to address engaging older adults with technology. A primary goal of UARC is to prevent social isolation and promote mental wellness through education. Using a hybrid model of videoconferencing and socially-distanced meetings, volunteer health professionals and community members (e.g., Information Technology Technician, Psychologist, and Nurse) delivered education to older adults. Participants also met for computer training to build confidence by sharing technology accomplishments with others (e.g., online bill paying). UARC has 75 active members and conducted over 12 videoconferences and in-person programming. The UARC project serves as a model for community/academic partnerships to support mental wellness and technology use in urban older adults.

